# Meta-analysis of muscle transcriptome data using the MADMuscle database reveals biologically relevant gene patterns

**DOI:** 10.1186/1471-2164-12-113

**Published:** 2011-02-16

**Authors:** Daniel Baron, Emeric Dubois, Audrey Bihouée, Raluca Teusan, Marja Steenman, Philippe Jourdon, Armelle Magot, Yann Péréon, Reiner Veitia, Frédérique Savagner, Gérard Ramstein, Rémi Houlgatte

**Affiliations:** 1 INSERM, U915, Nantes, F-44000 France; 2 Université de Nantes, Faculté de Médecine, Nantes, F-44000, France; 3 CHU de Nantes, l'Institut du Thorax, CIC, Nantes, F-44000, France; 4 CHU de Nantes, Laboratoire d'Explorations Fonctionnelles, Nantes, F-44000, France; 5 CHU de Nantes, Centre de Référence des Maladies Neuromusculaires Rares de l'Enfant et de l'Adulte Nantes-Angers, Nantes, F-44000, France; 6 Institut Jacques Monod, UMR7592-CNRS, Paris, F-75013, France; 7 Université Paris Diderot-Paris 7, Paris, F-75013, France; 8 INSERM, UMR 694, Angers, F-49033, France; 9 Université d'Angers, Angers, F-49033, France; 10 CHU Angers, Laboratoire de Biochimie et Biologie moléculaire, Angers, F-49033, France; 11 Laboratoire d'Informatique de Nantes Atlantique LINA, Ecole Polytechnique, Nantes, F-44000, France

## Abstract

**Background:**

DNA microarray technology has had a great impact on muscle research and microarray gene expression data has been widely used to identify gene signatures characteristic of the studied conditions. With the rapid accumulation of muscle microarray data, it is of great interest to understand how to compare and combine data across multiple studies. Meta-analysis of transcriptome data is a valuable method to achieve it. It enables to highlight conserved gene signatures between multiple independent studies. However, using it is made difficult by the diversity of the available data: different microarray platforms, different gene nomenclature, different species studied, etc.

**Description:**

We have developed a system tool dedicated to muscle transcriptome data. This system comprises a collection of microarray data as well as a query tool. This latter allows the user to extract similar clusters of co-expressed genes from the database, using an input gene list. Common and relevant gene signatures can thus be searched more easily. The dedicated database consists in a large compendium of public data (more than 500 data sets) related to muscle (skeletal and heart). These studies included seven different animal species from invertebrates (*Drosophila melanogaster, Caenorhabditis elegans*) and vertebrates (*Homo sapiens, Mus musculus, Rattus norvegicus, Canis familiaris, Gallus gallus*). After a renormalization step, clusters of co-expressed genes were identified in each dataset. The lists of co-expressed genes were annotated using a unified re-annotation procedure. These gene lists were compared to find significant overlaps between studies.

**Conclusions:**

Applied to this large compendium of data sets, meta-analyses demonstrated that conserved patterns between species could be identified. Focusing on a specific pathology (Duchenne Muscular Dystrophy) we validated results across independent studies and revealed robust biomarkers and new pathways of interest. The meta-analyses performed with MADMuscle show the usefulness of this approach. Our method can be applied to all public transcriptome data.

## Background

Our understanding of muscle physiology has evolved through the years by extensive studies aimed at identifying molecular and physiological mechanisms involved in normal muscle function and disease. The emergence of microarrays in the early 1990 s paved the way for the expansion of this area of research. This technology reliably quantifies the expression levels of the transcripts, providing a snapshot of the activity of several tens of thousands of mRNAs simultaneously [1-3]. Gene expression analysis enables to identify biomarkers [4,5] and gene signatures [6,7] in human and animal models.

Gene expression studies in the field of muscle research have generally been carried out using a rather limited set of conditions and replicates. Therefore, experimental designs tend to focus on a few specific research questions [see e.g. [8]]. Microarrays have allowed the exploration of many fields on a genomic scale. For instance, the molecular diversity of muscle fiber types, the physiological plasticity and adaptation of muscle, as well as muscle atrophy, muscle disease and muscle pharmacogenomics [9-11].

In consequence, microarray data has accumulated rapidly. The transcriptome data can be found in dedicated [e.g. Public Expression Profiling Resource PEPR [12]] or generic databases [e.g. Gene Expression Omnibus GEO [13]]. Collecting the different microarray data sets for meta-analysis adds a new dimension to gene expression data analysis by combining a large set of experimental conditions [14]. The quality of any meta-analysis depends on the quality of the underlying data [15]. While considerable divergence across different microarray platforms has been observed in the past [16,17], their current accuracy and reproducibility [18,19] now enable reliable comparisons to be made today. Since the landmark study by Rhodes *et al*. [20], several recent meta-analysis studies [21-24] have led to important results particularly in the field of cancer research [25-28]. For a given pathology or tissue, meta-analysis yields robust lists of differentially expressed genes (or DEGs). In such a case, each set of data can be considered as an independent validation step [29-31] enhancing the signal-to-noise ratio [20-24]. In addition, new pathways - that could not have been previously identified in isolated data sets - can emerge from a meta-analysis [32,33]. Finally, when applied to different pathologies, meta-analyses bring to light interesting differences or similarities [34].

Performing such comparisons across different organisms appears to be a particularly promising approach [35-37] to better understanding of human diseases. Although differences exist [38], a careful meta-analysis between species can also reveal similarities [39-41]. The animal model can thus replicate some aspects of the human disease [42], yielding important insights into the pathogenic mechanisms [43]. Recently, Calura *et al*. [44] identified a common molecular pathway of atrophy in muscle of multiple species under diverse physiological conditions. This work demonstrates that such comparisons are possible and can be very useful in the field of muscle research. This was generalized by Jelier *et al*. [45] who systematically compared 102 muscle-related microarray data sets, based on lists of up- and down-regulated DEGs.

There is a substantial potential for novel discoveries by comparing (and associating) microarray studies. Doing so requires, however, a concerted effort to identify and remove obstacles from the routine mass comparison of microarray data. The objective is to make this amount of data accessible and comparable for the broad scientific community in the field of muscle research. Such databasing allows for a systematic comparison of the results from different studies in order to identify consistent expression patterns [46]. Notably, experimental researchers can interpret new data by exploring these biologically significant patterns. Based on this concept, several web tools have already been developed. They can be divided into two main groups: the first group aims to compare lists of DEGs, whereas the second analyses gene co-expression across data sets.

In the first group, two databases have emerged to host and quickly integrate the results of microarray experiments: LOLA (List Of Lists Annotated) [47] and L2L (List to List) [48]. LOLA and L2L both gather lists of published DEGs. They allow investigators to compare their own data to lists of DEGs from different platforms and species in order to identify underlying patterns. However, they are quite limited by the size of the database and the reliance upon the way the lists were created (e.g. heterogeneous processing of the studies). To solve this problem, other tools, based on the re-analysis of data sets, have been developed with varying degrees of success [see [49] for review]. A major problem was the low amount of meaningful raw data deposited in public databases [50]. A more advanced comparison strategy of significant gene lists was provided by Oncomine [51] and GeneChaser (GENE CHAnge browSER) [52]. Oncomine is a comprehensive and expertly annotated database of gene expression studies. The collection comprises 25,447 samples in 360 experiments taken from 40 cancer types. This tool facilitates the identification of DEGs between cancer and normal tissues or among different cancer subtypes across a large collection of microarray data. This system was successful in performing comparative meta-profiling to identify shared gene expression signatures. However, this feature does not appear to be accessible to the user. Likewise, GeneChaser [52] automatically re-annotated and analyzed 1,515 GEO data sets from 231 microarray types across 42 species. It performed 12,658 group-versus-group comparisons to identify biological and clinical conditions in which a set of genes is differentially expressed. This tool also provides statistical and graphical representations to interpret these data. Two variant strategies have also been developed, both using signed rank genes as the basis for DEG 'signatures' from a two-group comparison. The first one is a microarray database search algorithm in an application called the Connectivity Map (CMAP) [53]. It gathers a reliable but small number (564) of drug-related cancer signatures in ten cell lines and derived from one laboratory using a single microarray platform. However, signatures derived from other platforms were not demonstrated to work with CMAP. The second strategy called EXALT (EXpression signature AnaLysis Tool) [54] holds thousands of DEGs (16,181) extracted from a large formatted collection of microarray results from GEO and published cancer studies. This collection represents hundreds of different experiments on many different tissues and generated on multiple platforms. The statistical approach used by the authors is similar to that proposed by Rhodes *et al*. [30]. It performs statistical tests and then calculates a p-value for each probe, separately for each study, resulting in a list of statistically de-regulated genes for each data set. However, these DEG-based methods have clear caveats. They often use a single significance test to extract DEGs from all experimental designs, and significant genes are defined based on a two-group comparison strategy. Although they adhere strictly to the group design specified by the investigators, DEGs cannot always be extracted from microarray data sets. Some GEO [13] data sets do not have sufficient information to provide statically reliable results. Additionally, no signature can be produced if a comparison between two groups is not statistically significant. Finally, additional novel comparisons within a data set are not possible: the current GEO data structure does not provide a computable attribute to automatically identify this type of experiment or hypothesis. To this end, other comparison methods, based on co-expression analysis of genes, have been considered. It has been shown that a sufficiently large and diverse set of profiles obtained under various physiological conditions results in the identification of co-regulated transcript groups [55]. Gene co-expression is conserved across microarray data sets [22] and can be identified in a compendium of gene expression data [56]. This strategy yields the detection of modules of co-expressed genes which are either specific to one physiological condition or shared across a set of different physiological conditions [57]. This approach of cross-platform analysis of microarray data has allowed the unraveling of networks of transcription factors in yeast [24]. This work examined the expression patterns of co-expressed gene pairs or 'doublets' across multiple data sets to infer functional linkages. The search for doublets was used in the GAN (Gene Aging Nexus) tool to explore co-expressed gene pairs across 42 data sets related to age [58] and was also recently implemented in OncoMine [51]. Based on the results obtained by Lee *et al*. [22], the Gemma database and software system was likewise developed for the re-use and meta-analysis of gene expression.

We have taken into account the advantages of the two strategies previously described for microarray data analysis. On those grounds, we developed a tool that makes muscle transcriptome data meta-analysis easily accessible to any user. Specifically, we have built a database that gathers all the public microarray data related to muscle studies from GEO [13]. After a careful re-analysis of microarray data, clusters of co-expressed genes were identified in each data set. Converted into lists of genes, our tool allows the simultaneous comparison of all clusters independently of the platform used and the species studied. This comparison enables to identify:

i) robust signatures of a pathology or a treatment across several independent studies.

ii) sets of genes that may be similarly modulated in different disease states or following drug treatments.

iii) common sets of co-expressed genes between human and animal models.

In the remaining sections of this paper, we first present the MADMuscle tool. We show how the user can browse the microarray data related to muscle studies, examine the annotated clusters and compare his own gene list with the gene lists relative to all the clusters of the database. In the next section, we have developed two meta-analyses to demonstrate the usefulness of our tool.

## Construction and content

We developed the MADMuscle database and an associated software tool to improve the comparison of muscle-related expression data from various studies or organism(s). In addition to just collecting microarray data, MADMuscle involves an automatic re-normalization and re-analysis of all these data sets to identify clusters of co-expressed genes (see Additional File 1). These clusters are functionally annotated and displayed in simple, well-annotated lists of genes, using a universal identifier. These gene lists, supported by clusters, are the basis for comparison of microarray data by meta-analysis (see Additional File 2).

Currently, MADMuscle contains more than 4,400 clusters of co-expressed genes identified from 535 distinct data sets corresponding to a wide range of conditions, from normal to pathological (for detailed statistics on the content of the database, see **part 1 **of the Additional File 3). Among them, 1,247 clusters automatically identified as good quality clusters were used for meta-analysis (developed in section "Utility and Discussion").

In this section, we first describe how the database was built, then we present the method used for the extraction of clusters and finally the database interrogation (i.e. from a user-defined gene list, the tool provides a list of statistically similar clusters).

### Data Collection and Processing

#### Data retrieval

Currently, the MADMuscle database collects all transcriptome data sets related to muscle studies from the public repository Gene Expression Omnibus (GEO, http://www.ncbi.nlm.nih.gov/geo/) [13] of the National Center for Biotechnology Information (NCBI) at the National Institutes of Health (NIH). These data sets were identified using the following keywords: "muscle", "myo", "heart" or "cardio". This represents a total of 535 analyzed data sets (see Additional File 1) corresponding to 447 unique GEO series, 116 different microarray platforms and 7 different species (*Homo sapiens *Hs, *Mus musculus *Mm, *Rattus norvegicus *Rn, *Canis familiaris *Cf, *Gallus gallus *Gg, *Drosophila melanogaster *Dm, and *Caenorhabditis elegans *Ce).

#### Data re-normalization

Raw data from GEO correspond most of the time to already pre-processed and/or normalized data. This step thus aims at normalizing the pre-normalized microarray data to remove artefacts and to ensure that each entry of the database follows the same procedure (see details in **part 2 **of the Additional File 3 and also Additional File 4). For each data set the K-nearest neighbors method [59] was used to evaluate the missing values in the microarray data sets. Then, non-linear effects such as background or saturation were corrected by LOWESS [60], as previously described [61,62], using a channel by channel procedure [63], each array being individually normalized to the median profile of all arrays.

#### Hierarchical clustering

**Figure 1:**
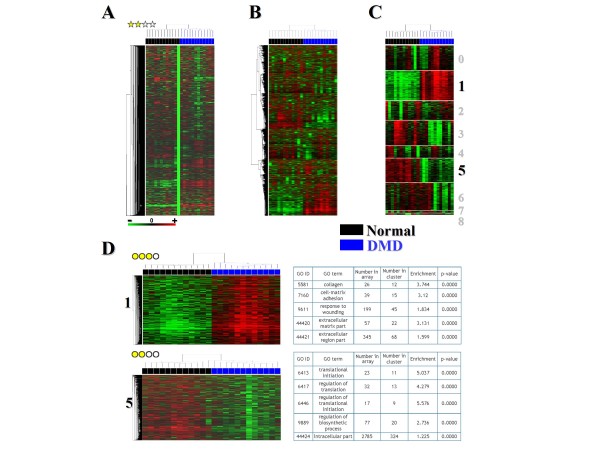
**Summary of the analysis performed for each data set of the MADMuscle database**. The estimated correct (two yellow stars) data set "*GSE1004_GPL91*" from Judith Haslett *et al*. [8], which explores molecular profiles of dystrophin-deficient (DMD, blue color) and normal (black color) human muscle, is used as an example. For every analyzed matrix, each row represents a gene, and each column represents a sample. Each cell in the matrix corresponds to an expression level, with red for over-expression, green for under-expression, and black for gene expression close to the median (see the color scale). Heat maps resulting from hierarchical clustering (genes and samples) of raw data from GEO (**A**) and re-normalized data (**B**) are given. The heat map of the distinct clusters (from 0 to 8) identified by the stable k-means procedure (**C**) is also displayed. After hierarchical clustering (genes and samples), data from each identified cluster (**D**) are also represented by a heat-map, given a quality estimation and functionally annotated. Among them, two clusters, which discriminate DMD from normal muscle, are exemplified (clusters 1 and 5). Cluster 1, estimated as a good cluster (three yellow dots), corresponds to 1,107 genes over-expressed in DMD muscle (DMD+) and is enriched in genes coding for proteins of the extracellular matrix. On the contrary, cluster 5, estimated as a correct cluster (two yellow dots), corresponds to 896 genes under-expressed in the DMD muscle (DMD-) and is enriched in genes coding for proteins involved in translation.

Hierarchical classification [55] was used to investigate relationships between samples and relationships between genes both on raw and re-normalized data (Figure 1A, B and Additional File 1). An average linkage clustering, using Pearson's correlation as similarity metric, was performed with Cluster 3.0 [64] and applied to data that were log-transformed and median-centred on genes [65].

### Cluster Analysis and Annotation

#### Cluster selection

Only expression matrixes with at least ten columns/samples (semi-empirically determined threshold) were kept for subsequent analysis (see **part 3 **of the Additional File 3 for detailed information). Clusters of co-expressed genes were identified using an iterative k-means [66] procedure (Figure 1C and Additional File 1) based on the Forgy's algorithm [67] and implemented in the statistical software package R [68]. Genes conserved in 95% of the 1000 independent k-means for a same cluster were retained (for a more detailed description of the method, see also **part 4 **of the Additional File 3). A total of 4,432 clusters of co-expressed genes were generated using the stable k-means procedure. An average two-way linkage clustering (i.e. genes and samples) was also applied to each of these clusters (Figure 1D and Additional File 1). The resulting heatmaps (hierarchical clustering and k-means) and trees were generated with the Linux command line clustergram image creation utility Slcview http://slcview.sourceforge.net.

#### Outlier detection in clusters

We defined an outlier as an aberrant gene expression value in one sample. We detected these aberrant values using a conventional method [69]. Considering a particular gene and its vector of expression values across samples, we defined Q1 and Q3 to be the first and third quartile of this vector. We defined IQR to be the interquartile range (Q3 - Q1), and the interval [Q1 - 1.5 × IQR, Q3 + 1.5 × IQR] was defined as the "normal" range of expression values. We thus considered outliers as gene expression values that lie outside this range. For a vector of normally distributed data, this threshold corresponds to approximately 5% of the data. For a particular sample, these aberrant expression values are often observed for numerous genes. K-means clustering is sensitive to such repeated aberrant values, leading to a high number of irrelevant gene signatures. To avoid such artifacts, we then identified for each gene signature, samples having gene expression values that are drastically different from the rest of the samples. In this context, samples with a proportion of outliers that exceeds 20% are rejected and the cluster is marked as "outlier".

#### Data set and cluster quality estimation

The quality of a cluster (Figure 1D and Additional File 1) is determined by a test statistic based on the Pearson's product-moment coefficient. Considering a pair of gene expression profiles, we computed the p-value from the Student's t-statistic used to test the null hypothesis of positive correlation. The quality *q *of the cluster *c *was defined as the geometric mean of the p-values of all the gene pairs belonging to *c*. For large clusters (more than 100 genes), *q *was estimated using a resampling technique: the computation was performed on a subset of 10,000 gene pairs randomly extracted from the cluster. Note that for clusters marked as "outlier", their quality was estimated from the remainder of the data set, excluding the samples with a proportion of outliers >20%. Five quality classes were defined as a function of the cluster p-value. The quality of a study (Figure 1A and Additional File 1) was inferred as the mean quality of its clusters (see **part 5 **of the Additional File 3 for more details on the quality classes associated to the clusters and to the studies).

#### Functional annotation of clusters

For each microarray platform, gene annotation was performed with the MADGene tool [70] (see also **part 6 **of the Additional File 3 for more details on this database). For each identified cluster of co-expressed genes, functional annotation (Figure 1D and Additional File 1) was performed using Gene Ontology (GO) [71] and GoMiner [72]. Significance of over- or under-representation of GO terms was calculated using Fisher's exact test.

### Database interrogation from a user-defined gene list

The similarity of a cluster with the gene list is based on a statistical comparison of the genes belonging to the clusters and the genes given by the user. This functionality aims at discovering the studies implying a specific set of co-expressed genes. The user can thus validate his gene list with previously published experiments (see Additional File 2).

#### Gene list re-annotation

The user can upload his gene list as a tab-delimited text file. Thanks to the MADGene resource [70] (see also Additional File 2 and **part 6 **of the Additional File 3), the user need not specify the identifier types that are used in his own list. When a list is submitted, the user can select the studied species and then the tool displays the gene annotation that has been performed for confirmation.

#### Cluster comparison

MADMuscle statistically assesses the overlap between the input gene list and the lists relative to all the clusters of the database (see Additional File 2). The concordance between the two lists is calculated as the number of genes in common divided by the number of genes in the input list. Although a single gene is allowed to occur multiple times in the same list, it is counted/considered only once in the comparisons. When the lists derive from different species, a gene from the input list is considered if its homolog is found in the cluster gene list, as reported in the NCBI HomoloGene database. MADMuscle performs pair-wise comparisons (Fisher's test) and produces a summary table that reports the number of common genes between the paired lists along with the p-value. For each comparison, the names of the common genes can be retrieved.

### Main characteristics of MADMuscle tool

MADMuscle gathers a large collection of muscle-related expression data sets from various studies or organism(s) (see Additional File 1 and **part 1 **of the Additional File 3). MADMuscle enables to perform meta-analysis of these transcriptome data by statistical comparison of gene lists supported by clusters of co-expressed genes (see Additional File 2). Indeed gene co-expression is conserved across many microarray data sets [22]. The databasing of such microarray gene lists thus allows for a systematic comparison of the results of various studies [48] in order to identify consistent expression patterns. It also helps experimenters to interpret new data in the context of these biologically significant patterns. For instance, this approach enabled Parmigiani *et al*. [73] to identify genes with consistent expression patterns across multiple lung cancer-related studies.

The comparison of heterogeneous platforms implies the conversion of the probe IDs into their corresponding approved symbols [74]. MADMuscle also takes into account the information on putative homologs between species. Once converted, gene lists can thus be compared whatever the microarray platform used or the species studied. Most of the published tools (e.g. Connectivity Map, L2L, Oncomine) rely on manual curation of data. Although these tools are extremely useful, they are labor-intensive. Thanks to the automatic microarray annotation tool MADGene [70] (see also Additional File 3, **part 6**), MADMuscle analyzes all muscle-related data sets from GEO in a fully automatic way. This frees users from the limitations of manually curated data sets, and facilitates the incorporation of new data.

MADMuscle is freely available and provides a simple interface for viewing, re-annotating, and comparing gene lists from clusters of co-expressed genes (for details about data storage and availability, see also Additional File 3, **part 7 **and **part 8**). The meta-analysis tool (see Additional File 2) highlights strong overlaps between any two gene lists. The tool compares each list in the database with the list of genes supplied by the user, and reports the statistical significance of any overlap between them. It also re-annotates each gene on the user's list with all the lists in the database on which it is found. The results are presented as a set of hyperlinked HTML documents, which can be conveniently explored by surfing from list to list and from gene to gene.

## Utility and Discussion

**Figure 2:**
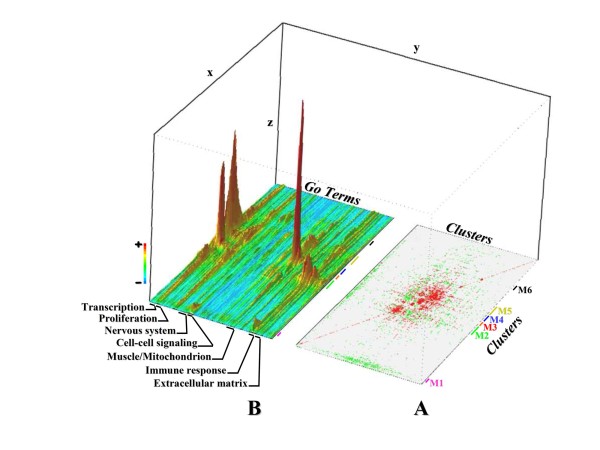
**Hidden patterns revealed by meta-clusters across muscle-related transcriptome data sets**. **A**- The right-hand side presents a systematic meta-analysis of all the "good" clusters from the MADMuscle database. The results are represented by the diagonally symmetric interaction matrix for 1,247 clusters of co-expressed genes based on statistical comparison (p-values). Red indicates significant similarity (strong overlap), green significant dissimilarity (poor overlap) and grey no overlap between compared clusters. The matrix was ordered using hierarchical clustering (lines and columns) to highlight groups of similar clusters. The resulting visualization uncovers 6 main components of highly similar and interconnected clusters, called meta-clusters (M1 to M6), involved in similar biological processes. **B**- On the left-hand side, a three-dimensional representation of the functional annotation of the meta-clusters is given. The functional annotation is visualized as a terrain map, in which highly correlated Gene Ontology (GO) terms are placed in proximity in the x-y plane and the probability density of enrichment in a region is shown by the altitude in the z direction. Each line represents a different GO term with cold colors for enrichment values <1 and hot colors for values >1 (see color scale). The rows (7554 GO terms) were ordered by hierarchical clustering, placing terms with similar annotation patterns together. The columns (clusters) were ordered in the same order as in the clustered diagonally symmetric interaction matrix (right-hand side of the figure). Specific significant GO annotation is associated to each Meta-cluster. For instance, the meta-cluster M4 supports genes encoding muscle and mitochondrion proteins.

**Figure 3:**
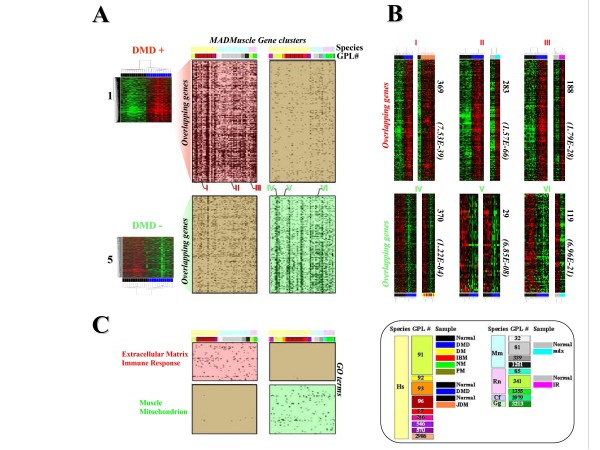
**Using the MADMuscle meta-analysis tool to perform systematic cross-species and cross-platform comparisons independently from the studied samples**. A- For each of the two input gene lists (clusters 1 and 5 presented in Figure 1), the 50 top overlapping clusters from the database were identified. Red and green backgrounds indicate significant overlaps with cluster 1 (DMD+) and cluster 5 (DMD-) respectively. Non-significant overlaps are displayed by a brown background. The 100 best-hit clusters, showing significant similarity with either cluster 1 or 5, cover 5 distinct species and 18 distinct microarray platforms (see color legend). **B**- Among the 100 top similar clusters, 3 particular results of overlapping genes are illustrated for cluster 1 (I, II and III) and 3 others for cluster 5 (IV, V, VI). For instance, 283 genes had increased expression (similarity p-value *p = 1.57e-66*) in both DMD muscle (input cluster 1) and mdx muscle (output cluster II). On the contrary, expression of 119 genes was commonly decreased (*p *= 6.96e-21) in both DMD (input cluster 5) and mdx (output cluster VI) muscles. **C**- Functional annotation of clusters 1 and 5 was inferred from their 50 best hits. For each of the 100 top clusters, the five top gene ontology (GO) terms are displayed using the same color code as depicted in part **A**. Cluster 1 resembles clusters enriched in genes coding for proteins of the extracellular matrix and proteins involved in immune response. Cluster 5 has similarity with clusters preferentially containing genes coding for muscle and mitochondrion proteins.

**Figure 4:**
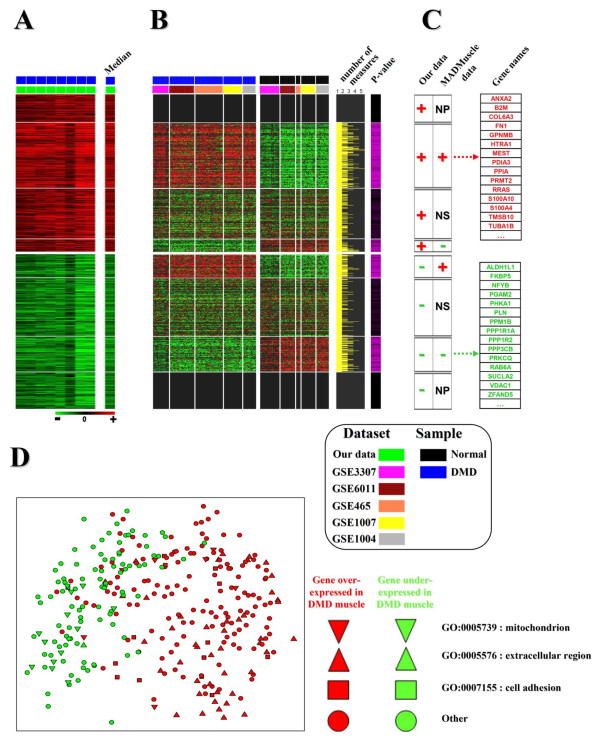
**Using the MADMuscle meta-analysis tool to refine gene expression patterns in a given pathology with a high level of confirmation**. A- Heat map showing the differentially expressed genes in DMD muscle from our data set. **B**- Heat map (merged overlapping clusters) of gene expression in normal and DMD muscle from five independent studies of the database. Samples (columns) were classified according to the experimental group (DMD and normal) and the data set of origin (see color legend). For each gene (rows), the yellow and purple bars indicate the total number of measurements (0 to 5) across studies and the computed p-values from the student's t-test. **C**- The comparison (**A **and **B**) identified 8 different classes of gene expression depending of their status in our data set ("+": over-expression; "-": under-expression) and across the other studies ("+/-": over/under-expression; "NS": no significant differential expression; "NP": no present in the clusters). Among them, the two classes **+**/**+ **and **-**/**-**, corresponding to genes commonly up- or down-regulated across all the DMD studies, are illustrated by some particular gene names (right hand side). **D**- Multidimensional scaling (MDS) [83] analysis of gene proximity (genes +/+ and -/-) given by the 1,247 "good" labeled clusters of the database. Results of the projection are represented by a 2 D plot in which green and red indicate gene commonly under and over-expressed in DMD muscle (classes -/- and +/+). Symbols (circle, square or triangle) illustrate particular Gene Ontology (GO) terms associated to these genes (see legend).

This section is divided into two major parts. The first one is a global analysis of the cluster database indicating its reliability (Figure 2). The second one is devoted to the demonstration of the utility and the relevance of the MADMuscle tool through the presentation of two meta-analyses related to the DMD (Duchenne Muscular Dystrophy) (Figure 3 and Figure 4).

### Meta-analysis to assess the reliability of the cluster database

#### Pairwise comparisons of the clusters

The objective was to identify common transcriptional programs of muscle development across a wide range of microarray data sets. Therefore, we systematically compared each cluster of the database to all others, using the statistical test presented in the section "*Cluster comparison*". Instead of one input gene list, we successively introduced all the gene lists contained in the cluster database. This pairwise comparison of clusters is illustrated in Figure 2. Each of the 1,247 "good" labeled clusters of the database was systematically compared to all others using the Fischer exact test to detect significant similarity (strong overlap) or dissimilarity (poor or no overlap). The resulting p-values were computed to generate the diagonally symmetric similarity matrix for the 1,247 clusters (Figure 2A).

Compiling large numbers of microarray studies in a single database simplifies the analysis as it integrates many conventional assays into a single genome-wide expression profile. Thus the results from different studies can be compared directly [23]. However, microarray studies often generate gene signatures consisting of hundreds of genes, making it difficult to distinguish which gene expression features are critical. For instance, in some data sets, a gene may show little variation and may have no co- expressed genes, whereas in other data sets the same gene may be part of a robust cluster of co-expressed genes [22]. In this type of situation, it is useful to compare the results of different studies - by means of meta-analysis - to determine which results are most robust and most consistent across a range of studies.

#### Identification of core meta-clusters

The result of our analysis is a global map showing the clusters that are shared under a wide variety of physiological conditions (Figure 2A). The classification of this map identified 6 major, distinct and wide meta-clusters (M1 to M6). Each of them contained at least 15 similar clusters from various studies, microarray platforms or species. Many other small meta-clusters, gathering less than 10 clusters, could also be identified but will not be discussed in the present study. Whereas M1 gathered 16 clusters, 79 clusters were found in M2, 25 in M3, 46 in M4, 59 in M5 and 19 in M6.

#### Functional annotation of core meta-clusters

It is likely that conserved co-expression patterns between studies have a functional relationship [22]. A sufficiently large and diverse set of profiles, obtained from various conditions, results in a relatively comprehensive identification of co-expressed transcript groups. This allows additional hypotheses to be drawn regarding the functions of genes based on the regulatory characteristics of their transcripts. Such an approach better reflects specific biological processes [55-57]. To assess the reliability of these meta-clusters, we evaluated their functional annotation by examining the overlap of GO terms for each cluster of co-expressed genes (Figure 2B). By taking into account both enrichment and p-values, we clearly showed that each of the identified meta-clusters is associated with specific GO terms. The functional annotation of the meta-clusters is discussed in **part 9 **of the Additional File 3.

This study enabled us to identify a transcriptional landscape of skeletal muscle. It correctly infers a number of known fundamental biological processes within a skeletal muscle context. We found that a substantial number of correlated expression patterns occur in multiple independent data sets (Meta-clusters). Since tight correlation may imply common regulation [75] the identification of the precise role of transcription factors (e.g. ESRRA) in the coordination of gene expression patterns will be followed by studies on muscle pathology as was recently done for other tissues [76].

### Meta-analysis to investigate the genomics of DMD muscle

In this part, we aim to demonstrate the utility and the relevance of the MADMuscle tool. With this objective in mind, we developed two analyses (Figure 3 and Figure 4) showing how easy and quick meta-analysis can produce novel biological insights from the large compendium of microarray data.

The first analysis illustrates the fact that conserved gene expression patterns can be identified between different pathologies and animal models. In this case, gene expression changes in different models are sufficiently similar to suggest a common underlying mechanism. To this end, two particular clusters (cluster 1 and 5, detailed in Figure 1) - discriminating normal from Duchenne Muscular Dystrophy (DMD) muscle in Haslett's data set [8]- were used as input gene lists and compared to the rest of the database (Figure 3 and **part 10 **of the Additional File 3).

The second analysis shows how robust biomarkers can be identified for a given pathology (see **part 11 **of the Additional File 3). Similar patterns of gene expression changes are easily found in several data sets to clearly define a set of pathology-related genes. Focusing on the DMD pathology, we first used the results from our own data set as input external gene lists (see **part 12 **of the Additional File 3). Then we retrieved the related studies from the database thanks to our query tool. Finally, we integrated them to identify robust biomarkers (Figure 4).

### Comparison of DMD with other diseases

#### Selection of two input gene lists

To illustrate the capacity of MADMuscle to perform such comparisons, we chose two clusters (Figure 1C and 1D) from the re-analyzed "GSE1004_GPL91" data set [8], which discriminate normal muscle from muscle affected by Duchenne Muscular Dystrophy (DMD). DMD is a severe genetic myopathy caused by the lack of the sarcolemmal protein dystrophin, and is clinically characterized by progressive and irreversible degeneration of muscle tissue. The first cluster (cluster 1, DMD+) contains 1,107 genes over-expressed in DMD muscle and involved in extracellular cell-matrix adhesion and the inflammatory-immune response (Figure 1D). The second cluster (cluster 5, DMD-) corresponds to 896 genes under-expressed in DMD muscle and involved in translation or coding for mitochondrion proteins (Figure 1D). Our results confirm the authors' initial observations [8] that many of the differentially expressed genes reflect changes in infiltration by inflammatory cells and connective tissue. Taken together, these two clusters recapitulate the major pathological feature of DMD which is abnormal connective tissue proliferation following myofiber degeneration.

#### Systematic meta-analysis of gene expression data

These histological observations, although dramatically exacerbated in the DMD muscle, should also be present in other pathological or physiological conditions. For instance, skeletal muscle fibrosis, along with connective tissue proliferation, is a major pathological hallmark of chronic myopathies. In these pathologies, myofibers are replaced by progressive deposition of collagen and other extracellular matrix proteins produced by muscle fibroblasts. It is thus reasonable to argue that the coordinated gene expression patterns that reflect these histological changes will be conserved across studies. Comparing genomic expression profiles across species can reveal evolutionary conserved mechanisms, as illustrated in McCarrol *et al*. [36]. Based on the MADMuscle meta-analysis tool, the 50 best hits for each of the two input gene lists (cluster 1 and cluster 5) were identified and analyzed (see Figure 3). These 100 clusters differ largely in their gene composition: 50 clusters display significant resemblance with the DMD+ cluster 1, while the 50 others contain significantly more genes observed in the DMD- cluster 5 (Figure 3A). Among the 100 top clusters showing significant overlaps with either cluster 1 or cluster 5, we identified 5 distinct species (*Homo sapiens *Hs, *Mus musculus *Mm, *Rattus Norvegicus *Rn, *Canis familiaris *Cf and *Gallus gallus *Gg), 18 distinct microarray platforms and various physiological conditions (including other muscle pathologies). This result underlines the capacity of the tool to highlight co-expressed genes across studies. We chose some typical examples among the 100 output results to illustrate this feature (Figure 3B). We found for instance that the two DMD gene signatures, namely DMD+ (cluster1) and DMD- (cluster 5), strongly resemble their counterparts (respectively II and VI: GSE466_GPL81) in the 16-wk-old mouse *mdx *muscle - the animal model of DMD - in spite of real discrepancies pointed out in the study [77]. Additional information can be found in **part 10 **of the Additional File 3.

#### Functional re-annotation

Finally, MADMuscle allows the identification of genes that change repeatedly in different studies, even when the studies are on different species or microarray platforms. The deregulation of these genes is caused by similar histological changes in the studied tissues. Reinforcing this idea is the fact that the GO terms (Figure 3C), supported by each of the 100 resulting clusters, fit very well with the direct functional annotation deduced from the two signatures DMD+ and DMD-. The GO terms associated with the 50 clusters resembling the DMD+ signature converged towards the extracellular matrix and the immune response; those associated with the 50 clusters resembling the DMD- signature identified muscle and mitochondrion markers. It is remarkable that functional annotation of the two signatures could thus have been deduced *a posteriori *from the meta-analysis. This may simplify the interpretation of lists of genes with altered expression, a critical and time-consuming part of microarray research.

### Comparison of DMD microarray studies

#### Meta-analysis to identify similar gene clusters

We used our own transcriptional analysis of muscle (fascia lata tensor or paravertebral) affected by DMD. This study is presented in **part 12 **of the Additional File 3. We obtained two lists of differential genes (Figure 4A): 483 genes were over-expressed (gene list "+") in the DMD muscle, 473 genes showed a clear down-regulation (gene list "-"). The two gene lists (+: up and -: down) from our study (Figure 4A) were computed using MADMuscle. Because of the conserved co-expression across studies [22], these two gene lists enabled us to retain clusters of co-expressed genes from 5 distinct independent GEO series related to DMD studies (Figure 4B) (GSE465 [78], GSE1004 [8], GSE1007 [79], GSE3307 [80], and GSE6011 [81]),

#### Integration of output data

The resulting clusters from each study were merged to create a meta-matrix (Figure 4B) in which genes differentially expressed between DMD and normal muscles were identified. The p-values can be calculated in each individual study and then combined, yielding an overall estimate of gene significance [23]. Another approach is to apply p-value combination only after the construction of a meta-profile, defined as the trimmed median expression profile of all the equally annotated features [82]. The combination of results from different studies partially solves the problem of a small sample number (inherent to microarray experiments) and thus helps to detect the truly differentially expressed genes. We therefore applied a Student's t-Test (p-value <0.01) on each gene expression meta-profile to identify significant variations between the control meta-group and the DMD meta-group. Reliably deregulated genes (meta-clusters of either up-regulated or down-regulated in DMD muscle) as well as invariant genes were identified.

#### Data validation

The results from the meta-matrix were confronted to those from our study (Figure 4C). As expected from the small number of samples explored in our data set, part of the results could not be validated (false positives). Actually, some DEGs were not present (NP) in MADMuscle clusters, indicating that they did not significantly vary in any data set (+/NP: 87 genes; -/NP: 113 genes). Other DEGs were not significant in most MADMuscle clusters (+/NS: 155 genes; -/NS: 179 genes). Some DEGs even varied significantly in the opposite sense to that observed in our study (+/-: 39 genes; -/+: 72 genes). These discrepancies in the results could reflect a muscle-type effect since in our study we used paravertebral and fascia lata tensor muscles whereas in the public data sets, mainly quadriceps muscle biopsies were investigated. On the contrary, 202 DEGs were found to be significantly up-regulated both in MADMuscle clusters and in our study (+/+ group) while 109 DEGs showed a clear down-regulation (-/- group).

Finally, to assess the differences between these two complete gene lists (+/+ and -/-), we analyzed their expression patterns, given by the 1,247 "good" labeled clusters of the database, with a Multidimensional scaling (MDS) [83]. This approach is particularly pertinent for the visualization of the similarities and differences observed in the data (Figure 4D and **part 13 **of the Additional File 3). The results, represented as a 2 D plot, show a clear separation between DMD+ (red) and DMD- (green) genes, achieved along one of the two components of the plot. One can note that DMD+ genes spread more widely than DMD- genes. This is probably due to the fact that in the DMD muscle, most of the up-regulated genes correspond to different invading cell types. Interestingly, genes from these two groups are also preferentially associated with specific GO terms (e.g. "mitochondrion" for DMD- genes; "extracellular region" and "cell adhesion" for DMD+ genes).

These observations are in agreement with the conclusions from previous studies on DMD muscle. In addition, among the perturbed biological functions, new interesting functions were identified by this meta-analysis and will be the scope of further studies. For instance, among the DMD+ genes, we clearly found an over-representation of GO terms associated with muscle (GO:0007517) and neuron (GO:0022008; GO:0048699) development. This could be explained by the following situation: while the satellite cell pool is quickly exhausted by repeated cycles of degeneration and regeneration [84], other resident muscle cell populations [85] may also contribute to muscle fiber regeneration, along with reinnervation [86,87] in the dystrophic muscle. These findings are now under investigation to clarify the precise role of these biomarkers in the context of the pathology.

## Conclusion

We have defined a new microarray meta-analysis tool named MADMuscle. Our methodology allows biologists to easily explore a large collection of microarray data related to muscle, through a user-friendly web interface with browse and search functions at multiple levels. While useful for microarray data comparison, MADMuscle is not limited to microarray results, and is equally capable of comparing results from other high-throughput technologies (SAGE, ChIP-on-chip or ChIP-seq, Protein-array and other proteomic analyses, large scale Real-time PCR, etc.). The only limitation is that the input data must correspond to a gene list. Finally, this work provides a simple and scalable framework for comparing and assessing the intersection of multiple gene expression signatures from disparate data sets. This approach will be increasingly useful as the mass of published transcriptome data grows. We are continually maintaining, improving and adding new functionalities to our tool. The database content will be updated once a year. We notably plan to include muscle transcriptome data sets from the ArrayExpress repository (EBI) in the next version of MADMuscle database. Moreover, the database and methods we describe here can form the basis for further large-scale explorations of gene expression data.

## Availability and requirements

The MADMuscle tool is freely available online from http://www.madtools.org, a web site dedicated to the analysis and annotation of DNA microarray data.

## Authors' contributions

DB initiated and supervised the study, performed the analysis and wrote the manuscript. ED and GR developed the methods. AB and RT developed the web site. AM and YP performed the experiment which was designed by RH. RV, FS, MS, PJ, GR and RH provided feedback and biological insight, and contributed to the design of the study and methods. All of the authors have read and approved the final manuscript.

## Supplementary Material

Additional File 1 **The MADMuscle web interface - database**. In this supplementary file, we present the database web interface with different screenshots. Click here for file

Additional File 2 **The MADMuscle web interface - meta-analysis tool**. In this supplementary file, we present the meta-analysis tool web interface with different screenshots. Click here for file

Additional File 3 **Supplementary material and results**. In this supplementary file, we give additional information and comments about the procedures and the results. Click here for file

Additional File 4 **Re- normalization of MADMuscle data sets**. In this supplementary file, we illustrate the re-normalization procedure of each data set to correct remaining bias. Click here for file
