# Supplementary material for: Meta-analysis of muscle transcriptome data using the MADMuscle database reveals biologically relevant gene patterns
Source: BMC Genomics. 2011 Feb 16;12:113. doi: 10.1186/1471-2164-12-113 (PMC3049149; doi:10.1186/1471-2164-12-113)
Supplement: Additional File 1 — The MADMuscle web interface - database. In this supplementary file, we present the database web interface with different screenshots. [file 1471-2164-12-113-S1.DOC]

**Additional file 1**: **The MADMuscle web interface - database**.

The different screenshots of pages from the MADMuscle web site show the results from a typical query to access the data sets and related analysis. **1**- The user must select the “Data sets” link. **2**- This immediately lists all the data sets stored and analyzed in the database. Each data set is described with a name (e.g. “GSE1004_GPL91” data set from Judith Haslett et al.), a title (e.g. “Molecular profiles of dystrophin-deficient and normal human muscle”), and a quality score (e.g. “p = 5.35e-2”) described with yellow stars (e.g. two yellow stars for a p-value “p = 5.35e-2”). **3**- To access a specific data set and related information, the user must click on data set’s name (e.g. “GSE1004_GPL91” data set). Results of hierarchical clustering, for raw GEO data (**4**), re-normalized data (**5**), and stable K-means are displayed (**6**). **7**- Clicking the “Selected clusters” button gives right to access the clusters (results of the stable k-means procedure) of co-expressed genes (e.g. “cluster 1”) (**8**), with quality estimation (e.g. three yellow dots corresponding to a “good cluster”) and their functional annotation. **9**- Gene lists can be accessed by clicking the “View gene list” button. **10**- Clicking the “Analysis tool” enables to perform meta-analysis of gene lists supported by clusters of co-expressed genes.
