# Supplementary material for: Meta-analysis of muscle transcriptome data using the MADMuscle database reveals biologically relevant gene patterns
Source: BMC Genomics. 2011 Feb 16;12:113. doi: 10.1186/1471-2164-12-113 (PMC3049149; doi:10.1186/1471-2164-12-113)
Supplement: Additional File 2 — The MADMuscle web interface - meta-analysis tool. In this supplementary file, we present the meta-analysis tool web interface with different screenshots. [file 1471-2164-12-113-S2.DOC]

**Additional file 2: The MADMuscle web interface – meta-analysis tool.**

The different screenshots of pages from the MADMuscle web site show the results from a typical query to perform a meta-analysis. **1**- The user must select the “Analysis tool” link to open the “Compare profiles” web page. **2**- The user’s gene list (cluster of co-expressed genes for instance) to be analyzed is loaded as the input file. **3**- The input species (e.g. “Homo sapiens”) must be selected in the drop-down menu. **4**- The “Test your list” button enables to give reliable annotation (Hugo symbol and full name notably) to your gene list, based on the MADGene database. **5**- The “compare” button enables to quickly perform a meta-analysis of the input cluster across all data sets, independently from the platform or the species. Results of the meta-analysis are sorted according to increasing p-values. **6**- For each data set from the results, the “list” button enables to display the overlapping genes between the input gene list and the cluster from the MADMuscle database.
