# Supplementary material for: Meta-analysis of muscle transcriptome data using the MADMuscle database reveals biologically relevant gene patterns
Source: BMC Genomics. 2011 Feb 16;12:113. doi: 10.1186/1471-2164-12-113 (PMC3049149; doi:10.1186/1471-2164-12-113)
Supplement: Additional File 3 — Supplementary material and results. In this supplementary file, we give additional information and comments about the procedures and the results. [file 1471-2164-12-113-S3.DOC]

**Additional file 3: Supplementary material and results.**

**#1# MADMuscle statistics**:

Currently, MADMuscle contains more than 4,400 clusters of co-expressed genes identified from 535 distinct data sets. Most of these data sets correspond to skeletal muscle (42%) and heart muscle (30%) studies. The major part of the data sets were human (34%), mouse (48%) and rat (14%) related studies. The top used microarray platforms are the GPL96 for human related studies (11% of the data sets), the GPL1261 (11%) for mouse related studies and the GPL85 (4%) for rat related studies. A text mining analysis of the GEO summaries from each data set identified the most studied conditions, ranging from normal to pathological. These conditions include muscle differentiation, muscle development, muscle remodelling, aging effect, stress effect, effect of various treatments (e.g. drugs, hormones), mutation effects and diseases (heart failure and dystrophies, including DMD), mitochondrial metabolism, hypertrophy and atrophy.

**#2# Re-normalization:**

Microarray data normalization is an important step for obtaining data that are reliable and usable for subsequent analysis. There are many sources of systematic variation in microarray experiments, e.g. different dye labelling efficiencies, scanner malfunctioning, differences in DNA concentrations on arrays (plate effects), printing or tip problems, uneven hybridization, background effect and saturation [1,2]. Normalization is the process of removing such variations. Whereas questions remain about the quality and effectiveness of normalization procedures, it is a necessary step that almost universally agreed upon in the scientific community [3].

Given the huge amount of data processed by GEO curators it is impractical to determine the quality and efficiency of the normalization methods used [4]. Although some of the data sets are raw data, the others correspond to pre-processed and/or normalised data sets.

For instance, the Affymetrix GeneChip® arrays are the most widely used in biological and medical research to estimate gene expression levels. In order to estimate gene expression values and perform high-level analyses, such as classification and clustering, probe-level pre-processing of these data is necessary. Typically, there are three steps of pre-processing: background correction, normalization and summarization, although not necessarily in that order. Some software packages allow for instance the user to interchange background correction methods with the normalization and summarization methods (e.g. Bioconductor [5]). One of the most popular methods to pre-process the Affymetrix data is the Robust Multichip Average (RMA). This method uses: an exponential-normal convolution model for background correction, a median polish algorithm to summarize probe level values into a single expression value per gene, and a quantile normalization for the normalization step [6]. A wide range of normalization procedures for high-density oligonucleotide gene expression array data is available today, ranging from global or local scaling to more sophisticated methods using semi-parametric or non-parametric statistical models [see e.g. [7-10]]. However, the quantile method remains popular because it was shown to have superior performance in term of bias, variance and computational efficiency, compared to several other methods [11].

*De novo* analysis of pre-processed Affymetrix muscle data sets can detect remaining biases in some data matrix (see also **supplemental Figure 3** for illustrations). Some samples exhibit a clear linear bias compared to others within a data set. For instance, in the study from Haslett *et al*. [12], one sample (GSM15832) of the GSE1004_GPL91 data set showed weaker global signal intensity than the others. Other samples also show regional non linear bias. For instance, in the study from von der Hagen *et al*. [13], the GSM50581 sample of the GSE2629_GPL81 data set exhibited a clear non-linear bias of the strongest values.

Since improper normalization can lead to incorrect conclusions or unacceptably high false-positive or false-negative rates, we systematically re-normalize all the data sets to correct the remaining biases. To this end, we developed a LOWESS (locally weighted scatterplot smoothing) normalisation procedure adaptable to all type of data sets (mono- and two-channel). The LOWESS algorithm [14] is one of the most commonly used normalization techniques and robust regression techniques, as found in the R version of lowess [15], are relatively insensitive to outliers. Although initially developed for the dual-channel cDNA arrays, we modified the initial method using a channel by channel procedure as previously described by Workman *et al*. [16]. In this procedure, for a specified data set, each sample is normalised close to the median profile of all the samples of the data set. Linear biases are thus corrected forcing array distributions to have the same central tendency and non linear biases are corrected locally fitting the distribution curve of the signal values to that of the median profile. Our method has been successfully already used to normalize one-channel [17] and two-channel [18] microarray data sets.

**#3# Minimum sample size:**

The sample size is the only parameter of the design of a study that is under the experimenter's control. It is well established that biological replication is essential [19]. However, many microarray studies are often limited to small sample sizes notably due to expense, time or logistics. The required number of samples for a microarray study depends largely on the relevant parameters: the biological variability of the samples within each class, the fold changes in expression that are desired to be detected, the detection sensitivity of the microarrays, and the acceptable error rates of the results [20]. Several methods have been put forward to address the optimal number of replicates [e.g. [20-25]] as well as for estimating sample sizes for classification studies [e.g. [26,27]]. These power analyses often indicate that larger sample sizes are warranted [28] since increased sample size improves both the accuracy and significance of classification results [29]. For instance, these studies suggest that a doubling in sample size could potentially increase the power of differential expression detection by fourfold. Using information from public databases improves this detection [30], notably the integration of multiple data sets as we performed with MADMuscle.

One of the most important and most common goals of microarray studies is to compare two groups (or more) of patients. For instance, it is possible to compare the transcriptome of healthy vs diseased individuals [31], treated vs untreated patients [32] or those of long- vs short-term survival patients [33], etc. Such comparisons may lead to new potential ways of diagnosis or even therapy [34,35] but they require careful design of the experiment, explicit hypothesis formulation, and an adequate sample size to obtain valid conclusions. However, for such common designs, in which two groups of cases are evaluated for differential expression, evidence indicates that a minimum of 5 biological cases per group should be analysed [see for e.g. [36-40]]. When 16 public datasets, mostly from cancer studies, were examined using a repeated sampling approach [41], it was observed that stable results for differentially expressed genes were not obtained until at least 5 biological replicates are used and that 10-15 replicates are needed for sufficient stability, which is also consistent with the results obtained by others similar studies [42,43]. One can note that this number is not the optimum since it only permits reliable detection of small subsets of the differently expressed genes [25,44].

Hierarchical clustering and other similar methods [45] have been shown to be effective in microarray data analysis for identifying genes with similar profiles and possibly with similar functions. However, in gene expression data analyses, the problem of a relatively small sample size is compounded by the very high dimensionality of the data available, making the clustering results especially sensitive to noise and susceptible to over-fitting. Sample sizes typically used in microarray research may be too small to support derivation of reliable clustering results [46] and can lead to artefactual results like heterogeneous clusters [47]. The reason is that the small number of samples results in unreliable correlation coefficients. As for the search for differential expression (previous paragraph) the correlation noise decreases as the samples size increases so that large sample sizes facilitate the identification of “true” coexpression. Although sample size calculations differ for differential expression and cluster analysis, Wu *et al.* [48] demonstrated that similar clustering outputs are obtained for studies having at least 9-10 samples, depending on the data set. This is in agreement with the fact that our method of gene cluster detection was not efficient when applied to data sets from MADMUscle database with less than 10 samples.

**#4# Cluster selection**:

Clusters of co-expressed genes were identified using an iterative k-means [49] procedure based on the Forgy’s algorithm [50] and implemented in the statistical software package R [15]. For each renormalized data set, 1000 independent k-means, using different random initial locations of the centroids, were applied (minimal number of genes: 50, minimal number of nodes: 9, maximum number of iterations per k-means: 1000) on normalized gene values (mean = 0 and standard deviation = 1) using Euclidian distance as (dis)similarity measure. A matrix *m* of co-occurrences was computed for each pair of genes. The element matrix *m_i,j_* corresponds to the frequency for which genes *g_i_* and *g_j_* are present in the same cluster. This matrix was used to define a graph *G* of co-occurrences. The vertices of *G* correspond to the genes; the edges denote the pair of genes having a co-occurrence frequency over a predefined threshold τ = 0.95. The extracted clusters were given by the connex components of *G*.

**#5# Data set and cluster quality estimation:**

Five quality classes were defined. Clusters with a p-value <0.001 were labeled as “very good” clusters; when 0.001<p<0.01, clusters were labeled as “good” clusters; when 0.01<p<0.05, clusters were labeled as “correct” clusters; when 0.05<p<0.2, clusters were labeled as “bad” clusters; and clusters with a p-value>0.2 were labeled as “noisy” clusters. Among the 4,432 generated clusters of co-expressed genes, 616 were labeled as “very good” clusters, 631 as “good”, 1,298 as “correct”, 1,275 as “bad” and 672 as “noisy”.

The quality of a study was inferred as the mean quality of its clusters. Likewise, data sets were defined as “very good” (p<0.01), “good” (0.01<p<0.05), and “correct (p>0.05). “Poor” quality data sets are those having less than 10 samples and were not analyzed. “Noisy” data sets are those having problems with one or more samples or exceeding missing values and could not have been analyzed. Among the 535 re-analyzed data sets, 101 (19%) were identified as having a quality mark of 4 (“very good”), whereas 107 (20%) had a quality mark of 3 (“good”), 126 (24%) a quality mark of 2 (“correct”), 152 (28%) a quality mark of 1 (“bad”) and 49 (9%) a quality mark of 0 (“noisy”).

**#6# Gene Annotation**:

For each microarray platform, information on probe-sets was gathered in the MADGene database [51] and completed with related information collected from the NCBI Entrez gene database [52]. Information on putative homologs of candidate genes from 17 different organisms, as well as transcript sequences from the same transcription locus were collected from the NCBI Homologene [53] and Unigene [54] databases.

Currently, the MADGene database supports 13 different identifiers (official symbols, synonyms, Genbank accession numbers, RefSeq IDs, Unigene cluster IDs, EntrezGene IDs, clone IDs, Ensembl gene and transcript IDs, Uniprot IDs, Affymetrix, Agilent and Illumina probe sets among all). The 17 following species are considered: *Anopheles gambiae*, *Arabidopsis thaliana*, *Bos taurus*, *Caenorhabditis elegans*, *Canis familiaris*, *Drosophila melanogaster*, *Danio rerio*, *Gallus gallus*, *Homo sapiens*, *Magnaporthe grisea*, *Mus musculus*, *Neurospora crassa*, *Oryza sativa*, *Pan troglodytes*, *Plasmodium falciparum*, *Rattus norvegicus*, and *Saccharomyces cerevisiae*.

**#7# Data storage**:

MADMuscle employs a three-tier web application architecture. In tier 1, data (raw, processed and analyzed) are stored in the relational database management system (RDBMS) MySQL. The tier 2 application layer is implemented in Hypertext Preprocessor (PHP, www.php.net), which is executed via calls from the tier 3 Apache web server running on the Linux operating system.

**#8# Data availability**:

MADMuscle requires registration and is freely available for browsing, uploading of lists, and comparing publicly available gene lists. All the data are freely available and downloadable via a web-based interface ([http://www.madtools.org](http://cardioserve.nantes.inserm.fr/madtools/home/)). For each data set, raw data, re-normalized data, results of stable k-means procedures, clusters and their related gene lists and functional annotation are displayed.

**#9# Functional annotation of core clusters:**

Six major meta-clusters (M1 to M6) could be identified. Some of them (M1, M3 and M5) correspond to relatively general signatures involved in glucose transport and cell-cell signaling (M1, GO: 0015758 and GO: 0007267), cell proliferation (M3, GO: 0051301 and GO: 0000278) and transcription (M5, GO: 0006350 and GO: 0006355). The others (M2, M4 and M6) correspond to more specific signatures regarding muscle physiology. M2 is associated with a signature of the pathological state of the muscle and contains genes involved in the inflammatory (GO: 0006954), immune (GO: 0002376) and defense (GO: 0006952) responses. This signature corresponds well with the symptoms, namely inflammatory response, necrosis and fibrosis, observed in most of the muscle pathologies. M4 exhibits a very specific muscle signature, gathering genes involved in the muscle system process (GO: 0003012). This signature includes proteins of the myofibril (GO: 0030016), notably those involved in striated muscle contraction (GO: 0006941), as well as proteins of the sarcomere (GO: 0030017). Genes coding for proteins of the mitochondrion (GO:0005739), particularly those of the mitochondrial respiratory chain (GO:0005746) involved in adenosine triphosphate (ATP) biosynthesis (GO:0042773), were also found to be closely associated with this muscle signature. Interestingly, the transcription factor ESRRA, that has been shown to target a set of promoters involved in the uptake of energy substrates, production and transport of ATP across the mitochondrial membranes, and intracellular fuel sensing, as well as Ca2+ handling and contractile work [55], was found to be part of this signature. M6 corresponds to an unexpected signature of the neuromuscular junction. Many of the M6 genes were found to be involved in cell junction (GO: 0030054) and nervous system development (GO: 0007399), neurological system processes (GO: 0050877), transmission of nerve impulses (GO: 0019226) or synapse (GO: 0045202) and synaptic transmission (GO: 0007268). Skeletal muscles express a large number of genes that allow the transmission of signals from neurons through the neuromuscular synapse. The formation of this structure occurs through exchange of information between neuron and muscle [56] through myogenic regulatory factors. These latter direct the assembly and usage of the neuromuscular junction [57].

**#10# Systematic meta-analysis of gene expression data:**

Among the 100 output lists from the meta-analysis, some typical examples were chosen to illustrate the pertinence of the results. We found for instance that the two DMD gene signatures, namely DMD+ (cluster1) and DMD- (cluster 5), strongly resemble their counterparts (respectively II and VI: GSE466_GPL81) in the 16-wk-old mouse mdx muscle - the animal model of DMD - in spite of real discrepancies pointed out in the study [58]. In the mouse mdx muscle, the cluster of down-regulated genes, encoding myofibril and mitochondrial proteins, significantly overlapped with the DMD- gene signature (VI: n=119, p-value=6.96E-21). Over-expressed genes, which code for proteins involved in the immune response (particularly the NF-kappaB cascade) and extracellular matrix, were very similar to the DMD+ signature (II: n=283, p-value=1.57E-66).

According to this DMD+ signature (cluster1), similar gene over-expression (I: GSE11971_GPL96; n=369, p-value=7.53E-39) could be observed in muscle of untreated children with juvenile dermatomyositis (JDM). These patients show chronic inflammation associated with dendritic cell maturation and anti-angiogenic vascular remodeling, directly contributing to disease pathophysiology [59]. Significant overlap (III: GSE4105_GPL341; n=188, p-value=1.79E-28) was also found in the rat heart, during the inflammatory phase of ischemia-reperfused (IR) remodeling myocardium [60].

Conversely, the DMD- signature (cluster 5), which shares high similarity with that observed in the mouse mdx muscle (VI: n=119, p-value=6.96E-21), also resembles a cluster of down-regulated genes in muscle biopsies affected by inflammatory myopathies (IV: GSE2044_GPL91; n=370, p-value= 1.22E-84) [61]. These results reflect muscle loss by degeneration along with a generalized mitochondrial dysfunction and “metabolic crisis” [62]. Far from being the primary cause of the disease, mitochondrial-dependent apoptosis and necrosis also represents a prominent disease mechanism in muscular dystrophy and may thus largely contribute to amplify the symptomatic fate of the muscle. In addition, these results are confirmed by another independent data set related to DMD (V: GSE1007_GPL93; n=29, p-value= 6.85E-08) [63].

**#11# Meta-analysis of gene expression data related to the same pathology:**

MADMuscle enables to evaluate, integrate, and inter-validate multiple data sets, with the ultimate goal of identifying biomarkers of the studied pathology.

Because of the explosion of the use of microarray technology, several research groups have conducted gene expression profiling studies in the same research area (same pathology). However, if one group finds a DEG (Differentially Expressed Gene) using microarrays, there is a risk that this is simply a false positive. If two groups independently find that the same gene is differentially expressed, the risk of this error is reduced. By combining results across several microarray experiments, we can therefore significantly improve the detection of true DEGs. This approach thus avoids the use of often expensive and time-consuming traditional laboratory methods (e.g. quantitative RT-PCR, northern or western blots, tissue microarrays) to determine true markers.

Simplifying the meta-analysis of multiple data sets addressing a similar hypothesis was one of the guiding factors behind the development of the MADMuscle tool. The objective is to bioinformatically validate and statistically assess all of the positive results simultaneously. Applied to DMD muscle studies, we demonstrate that five public data sets share significantly similar results, validating our own study. Beyond the specific implications for DMD, our method establishes a much-needed model for the evaluation, cross-validation, and comparison of multiple profiling studies.

**#12# Transcriptional analysis of muscle affected by Duchenne Muscular Dystrophy (DMD):**

We carried out gene expression analysis of 4 duplicated DMD samples and related controls. Muscles from four DMD patients (ages: 11-13 years) undergoing orthopedic surgery were compared to a reference constituted from muscles from seven young adults undergoing surgery for idiopathic scoliosis. Muscular biopsies (paravertebral: 1; fascia lata tensor: 3) were surgery by-products obtained in compliance with current ethical and legislative rules. The transcriptome experiment was performed using a previously published protocol [64-67].

***Study design*.** Each of the 4 DMD samples was compared to a common reference sample consisting of a pool of equal quantities of mRNA from muscles from 7 young adults. This complex mRNA pool was used as a standard or a common point of measurement that enabled a comparison between the individual DMD mRNA samples.

Control of technical and biological noises inherent to microarray experiments was incorporated in the study design. To account for the technical fluctuation of the expression measurements, eight technical replicate values were obtained for each DMD sample. To determine the fluctuation due to tissue sampling, two biological replicate samples were obtained from each DMD biopsies.

***Microarrays*.** Microarrays were prepared in-house using human-specific 50-mer oligonucleotide probes (MWG Biotech®). The probes were spotted onto epoxy-silane coated glass slides using the Lucidea Array Spotter (Amersham®). The 4217 human genes that were represented on the microarray had been selected for involvement in cardiovascular and/or skeletal muscle normal and pathological functioning. Selection was based on subtractive hybridization experiments [68,69], genome-wide microarray hybridizations [70] and literature data. Each probe was spotted in quadruplicate. For more information see: <http://cardioserve.nantes.inserm.fr/genomique_integrative/spip.php?article59>

***RNA isolation, labeling, and hybridization*.** Total RNA was isolated using TRIZOL® reagent (Life Technologies). mRNA was isolated using the Oligotex mRNA kit (Qiagen). RNA and mRNA quality was assessed using an Agilent 2100 bioanalyzer. Cy3- and Cy5-labeled cDNA was prepared using the CyScribe cDNA Post Labeling Kit (Amersham Pharmacia Biotech). Each individual mRNA sample was Cy5-labeled and mixed with an equal amount of Cy3-labeled reference sample. The mixture was pre-incubated with human Cot-I DNA (Gibco-BRL), yeast tRNA, and polyA RNA, and hybridized onto duplicate microarrays.

***Raw data extraction and consolidation*.** Hybridized arrays were scanned by fluorescence confocal microscopy (Scanarray 4000XL, GSI-Lumonics). Fluorescence signal measurements were obtained separately for each fluorochrome at 10 μm/pixel resolution. Hybridization and background signal intensities, and quality control parameters were measured using GenePix Pro 5.0 (Axon®). Analysis of the expression level was performed using the “MicroArray Data Suite of Computed Analysis” (MADSCAN, <http://cardioserve.nantes.inserm.fr/mad/madscan/>) automated procedure [71]: values corresponding to flawed spots were flagged and data were normalized by lowess. Genes of which all signal intensities were below the background level were filtered out. For each microarray, expression values were calculated as log2 (Cy5/Cy3). For each biological sample, expression values were then consolidated as the median of the eight technical replicate values.

***Statistical analysis of the data*.** After normalization and consolidation of the expression data with MADSCAN [71], differentially expressed genes were identified using a statistical strategy based on one-class Significance Analysis of Microarrays (SAM) [72] and LInear Models for MicroArray data (Limma) [73]. A consistent list of 956 genes was found to be differentially expressed in dystrophic muscles compared with the reference (FDR<0.02, p<0.01). Whereas 483 genes were over-expressed (gene list “+”) in the DMD muscle, 473 genes showed a clear down-regulation (gene list “-“). Functional annotation highlighted some physiological pathways (e.g. those involving matrix metalloproteinases and their inhibitors, the TGFβ pathways and calcium metabolism) that were altered in our study and also in other data sets.

**#13# Multidimensional scaling (MDS) analysis:**

The meta-analysis of 6 independent data sets enabled us to identify 311 genes whose expression was reliably deregulated in the DMD muscle. Among them, 202 genes were found to be significantly up-regulated both in MADMuscle clusters and in our study (+/+ group) while 109 genes showed a clear down-regulation (-/- group).

To assess the differences between these two complete and robust gene lists (+/+ and -/-) in term of gene proximity, we analyzed their expression patterns given by the 1,247 “good” labeled clusters of the database. To evaluate similarity (or dissimilarity), a pseudo-distance metric, based on the p-value that works as similarity measure, was designed to estimate the proximity of two genes (namely *g1* and *g2*) across all “good” clusters. P-value was computed from the hypergeometric distribution to find *g1* and *g2* in the same set of clusters, knowing the total number of clusters where *g1* appears, as well as the total number of compatible clusters. By ‘compatible’ we mean that we took in consideration the actual occurrence of both *g1* and *g2* in the microarray platform relative to each cluster. As the p-value tends to provide very low values, the pseudo-distance was set to the negative value of the logarithm of the p-value. The high dimensionality of the gene expression data was reduced to 2 dimensions (the two axes of the plot) that comprise the greatest variation across the data set. Results from the unsupervised multidimensional scaling (MDS) [74] projection were represented by a 2D plot in which the smaller the distance between genes, the greater their topological overlap

Reference List

1. Bilban M, Buehler LK, Head S, Desoye G, Quaranta V: **Normalizing DNA microarray data.** *Curr Issues Mol Biol* 2002, **4:** 57-64.

2. Quackenbush J: **Microarray data normalization and transformation.** *Nat Genet* 2002, **32 Suppl:** 496-501.

3. Lyons-Weiler J: **Profound normalisation challenges remain in the analysis of data from microarray experiments.** *Appl Bioinformatics* 2003, **2:** 193-195.

4. Barrett T, Troup DB, Wilhite SE, Ledoux P, Rudnev D, Evangelista C *et al*.: **NCBI GEO: mining tens of millions of expression profiles--database and tools update.** *Nucleic Acids Res* 2007, **35:** D760-D765.

5. Gentleman RC, Carey VJ, Bates DM, Bolstad B, Dettling M, Dudoit S *et al*.: **Bioconductor: open software development for computational biology and bioinformatics.** *Genome Biol* 2004, **5:** R80.

6. Irizarry RA, Hobbs B, Collin F, Beazer-Barclay YD, Antonellis KJ, Scherf U *et al*.: **Exploration, normalization, and summaries of high density oligonucleotide array probe level data.** *Biostatistics* 2003, **4:** 249-264.

7. Fan J, Chen Y, Chan HM, Tam PK, Ren Y: **Removing intensity effects and identifying significant genes for Affymetrix arrays in macrophage migration inhibitory factor-suppressed neuroblastoma cells.** *Proc Natl Acad Sci U S A* 2005, **102:** 17751-17756.

8. Li C, Wong WH: **Model-based analysis of oligonucleotide arrays: expression index computation and outlier detection.** *Proc Natl Acad Sci U S A* 2001, **98:** 31-36.

9. Schadt EE, Li C, Ellis B, Wong WH: **Feature extraction and normalization algorithms for high-density oligonucleotide gene expression array data.** *J Cell Biochem Suppl* 2001, **Suppl 37:** 120-125.

10. Sidorov IA, Hosack DA, Gee D, Yang J, Cam MC, Lempicki RA *et al*.: **Oligonucleotide microarray data distribution and normalization.** *Information Sciences* 2002, **146:** 67-73.

11. Bolstad BM, Irizarry RA, Astrand M, Speed TP: **A comparison of normalization methods for high density oligonucleotide array data based on variance and bias.** *Bioinformatics* 2003, **19:** 185-193.

12. Haslett JN, Sanoudou D, Kho AT, Bennett RR, Greenberg SA, Kohane IS *et al*.: **Gene expression comparison of biopsies from Duchenne muscular dystrophy (DMD) and normal skeletal muscle.** *Proc Natl Acad Sci U S A* 2002, **99:** 15000-15005.

13. von der Hagen M, Laval SH, Cree LM, Haldane F, Pocock M, Wappler I *et al*.: **The differential gene expression profiles of proximal and distal muscle groups are altered in pre-pathological dysferlin-deficient mice.** *Neuromuscul Disord* 2005, **15:** 863-877.

14. Yang YH, Dudoit S, Luu P, Lin DM, Peng V, Ngai J *et al*.: **Normalization for cDNA microarray data: a robust composite method addressing single and multiple slide systematic variation.** *Nucleic Acids Res* 2002, **30:** e15.

15. Ihaka R, Gentleman R: **R: A Language for Data Analysis and Graphics.** *Journal of Computational and Graphical Statistics* 1996, **5:** 299-314.

16. Workman C, Jensen LJ, Jarmer H, Berka R, Gautier L, Nielser HB *et al*.: **A new non-linear normalization method for reducing variability in DNA microarray experiments.** *Genome Biol* 2002, **3:** research0048.

17. Baron D, Montfort J, Houlgatte R, Fostier A, Guiguen Y: **Androgen-induced masculinization in rainbow trout results in a marked dysregulation of early gonadal gene expression profiles.** *BMC Genomics* 2007, **8:** 357.

18. Lamirault G, Le MN, Roussel JC, Le Cunff MF, Baron D, Bihouee A *et al*.: **Molecular risk stratification in advanced heart failure patients.** *J Cell Mol Med* 2009.

19. Allison DB, Cui X, Page GP, Sabripour M: **Microarray data analysis: from disarray to consolidation and consensus.** *Nat Rev Genet* 2006, **7:** 55-65.

20. Gadbury GL, Page GP, Edwards JW, Kayo T, Prolla TA, Weindruch R *et al*.: **Power analysis and sample size estimation in the age of high dimensional biology: a parametric bootstrap approach illustrated via microarray research.** *Statistical Methods in Medical Research* 2004, **13:** 325-338.

21. Hwang D, Schmitt WA, Stephanopoulos G, Stephanopoulos G: **Determination of minimum sample size and discriminatory expression patterns in microarray data.** *Bioinformatics* 2002, **18:** 1184-1193.

22. Muller P, Parmigiani G, Robert C, Rousseau J: **Optimal sample size for multiple testing: The case of gene expression microarrays.** *Journal of the American Statistical Association* 2004, **99:** 990-1001.

23. Pan W, Lin J, Le CT: **How many replicates of arrays are required to detect gene expression changes in microarray experiments? A mixture model approach.** *Genome Biol* 2002, **3:** research0022.

24. Pawitan Y, Michiels S, Koscielny S, Gusnanto A, Ploner A: **False discovery rate, sensitivity and sample size for microarray studies.** *Bioinformatics* 2005, **21:** 3017-3024.

25. Zien A, Fluck J, Zimmer R, Lengauer T: **Microarrays: how many do you need?** *J Comput Biol* 2003, **10:** 653-667.

26. Dobbin K, Simon R: **Sample size determination in microarray experiments for class comparison and prognostic classification.** *Biostatistics* 2005, **6:** 27-38.

27. Garge NR, Page GP, Sprague AP, Gorman BS, Allison DB: **Reproducible clusters from microarray research: whither?** *BMC Bioinformatics* 2005, **6 Suppl 2:** S10.

28. Wei C, Li J, Bumgarner RE: **Sample size for detecting differentially expressed genes in microarray experiments.** *BMC Genomics* 2004, **5:** 87.

29. Mukherjee S, Tamayo P, Rogers S, Rifkin R, Engle A, Campbell C *et al*.: **Estimating dataset size requirements for classifying DNA microarray data.** *J Comput Biol* 2003, **10:** 119-142.

30. Kim RD, Park PJ: **Improving identification of differentially expressed genes in microarray studies using information from public databases.** *Genome Biol* 2004, **5:** R70.

31. Beer DG, Kardia SL, Huang CC, Giordano TJ, Levin AM, Misek DE *et al*.: **Gene-expression profiles predict survival of patients with lung adenocarcinoma.** *Nat Med* 2002, **8:** 816-824.

32. Swagell CD, Henly DC, Morris CP: **Expression analysis of a human hepatic cell line in response to palmitate.** *Biochem Biophys Res Commun* 2005, **328:** 432-441.

33. Pass HI, Liu Z, Wali A, Bueno R, Land S, Lott D *et al*.: **Gene expression profiles predict survival and progression of pleural mesothelioma.** *Clin Cancer Res* 2004, **10:** 849-859.

34. Golub TR, Slonim DK, Tamayo P, Huard C, Gaasenbeek M, Mesirov JP *et al*.: **Molecular classification of cancer: class discovery and class prediction by gene expression monitoring.** *Science* 1999, **286:** 531-537.

35. Welsh JB, Zarrinkar PP, Sapinoso LM, Kern SG, Behling CA, Monk BJ *et al*.: **Analysis of gene expression profiles in normal and neoplastic ovarian tissue samples identifies candidate molecular markers of epithelial ovarian cancer.** *Proc Natl Acad Sci U S A* 2001, **98:** 1176-1181.

36. Allison DB, Gadbury GL, Heo M, Fernández JR, Lee C-K, Prolla TA *et al*.: **A mixture model approach for the analysis of microarray gene expression data.** *Computational Statistics & Data Analysis* 2002, **39:** 1-20.

37. Lin WJ, Hsueh HM, Chen JJ: **Power and sample size estimation in microarray studies.** *BMC Bioinformatics* 2010, **11:** 48.

38. Pavlidis P, Li Q, Noble WS: **The effect of replication on gene expression microarray experiments.** *Bioinformatics* 2003, **19:** 1620-1627.

39. Tsai CA, Hsueh HM, Chen JJ: **Estimation of false discovery rates in multiple testing: application to gene microarray data.** *Biometrics* 2003, **59:** 1071-1081.

40. Tsai CA, Wang SJ, Chen DT, Chen JJ: **Sample size for gene expression microarray experiments.** *Bioinformatics* 2005, **21:** 1502-1508.

41. Pavlidis P, Li Q, Noble WS: **The effect of replication on gene expression microarray experiments.** *Bioinformatics* 2003, **19:** 1620-1627.

42. Hwang D, Schmitt WA, Stephanopoulos G, Stephanopoulos G: **Determination of minimum sample size and discriminatory expression patterns in microarray data.** *Bioinformatics* 2002, **18:** 1184-1193.

43. Wang SJ, Chen JJ: **Sample size for identifying differentially expressed genes in microarray experiments.** *J Comput Biol* 2004, **11:** 714-726.

44. Gadbury GL, Page GP, Edwards J, Kayo T, Prolla TA, Weindruch R *et al*.: **Power and sample size estimation in high dimensional biology.** *Statistical Methods in Medical Research* 2004, **13:** 325-338.

45. Sherlock G: **Analysis of large-scale gene expression data.** *Curr Opin Immunol* 2000, **12:** 201-205.

46. Garge NR, Page GP, Sprague AP, Gorman BS, Allison DB: **Reproducible clusters from microarray research: whither?** *BMC Bioinformatics* 2005, **6 Suppl 2:** S10.

47. Segal MR, Dahlquist KD, Conklin BR: **Regression approaches for microarray data analysis.** *J Comput Biol* 2003, **10:** 961-980.

48. Wu FX, Zhang WJ, Kusalik AJ: **Determination of the minimum number of microarray experiments for discovery of gene expression patterns.** *BMC Bioinformatics* 2006, **7 Suppl 4:** S13.

49. Tavazoie S, Hughes JD, Campbell MJ, Cho RJ, Church GM: **Systematic determination of genetic network architecture.** *Nat Genet* 1999, **22:** 281-285.

50. Forgy EW: **Cluster Analysis of Multivariate Data: Efficiency vs. Interpretability of Classification.** *Biometrics* 1965, **21:** 768-780.

51. Baron D, Bihouee A, Teusan R, Dubois E, Savagner F, Steenman M *et al*.: **MADGene: retrieval and processing of gene identifier lists for the analysis of heterogeneous microarray datasets.** *Bioinformatics* 2011.

52. Maglott D, Ostell J, Pruitt KD, Tatusova T: **Entrez Gene: gene-centered information at NCBI.** *Nucleic Acids Res* 2007, **35:** D26-D31.

53. Wheeler DL, Church DM, Lash AE, Leipe DD, Madden TL, Pontius JU *et al*.: **Database resources of the National Center for Biotechnology Information.** *Nucleic Acids Res* 2001, **29:** 11-16.

54. Wheeler DL, Church DM, Lash AE, Leipe DD, Madden TL, Pontius JU *et al*.: **Database resources of the National Center for Biotechnology Information: 2002 update.** *Nucleic Acids Res* 2002, **30:** 13-16.

55. Dufour CR, Wilson BJ, Huss JM, Kelly DP, Alaynick WA, Downes M *et al*.: **Genome-wide orchestration of cardiac functions by the orphan nuclear receptors ERRalpha and gamma.** *Cell Metab* 2007, **5:** 345-356.

56. Burden SJ: **Building the vertebrate neuromuscular synapse.** *J Neurobiol* 2002, **53:** 501-511.

57. Blais A, Tsikitis M, costa-Alvear D, Sharan R, Kluger Y, Dynlacht BD: **An initial blueprint for myogenic differentiation.** *Genes Dev* 2005, **19:** 553-569.

58. Tseng BS, Zhao P, Pattison JS, Gordon SE, Granchelli JA, Madsen RW *et al*.: **Regenerated mdx mouse skeletal muscle shows differential mRNA expression.** *J Appl Physiol* 2002, **93:** 537-545.

59. Chen YW, Shi R, Geraci N, Shrestha S, Gordish-Dressman H, Pachman LM: **Duration of chronic inflammation alters gene expression in muscle from untreated girls with juvenile dermatomyositis.** *BMC Immunol* 2008, **9:** 43.

60. Roy S, Khanna S, Kuhn DE, Rink C, Williams WT, Zweier JL *et al*.: **Transcriptome analysis of the ischemia-reperfused remodeling myocardium: temporal changes in inflammation and extracellular matrix.** *Physiol Genomics* 2006, **25:** 364-374.

61. Greenberg SA, Sanoudou D, Haslett JN, Kohane IS, Kunkel LM, Beggs AH *et al*.: **Molecular profiles of inflammatory myopathies.** *Neurology* 2002, **59:** 1170-1182.

62. Chen YW, Zhao P, Borup R, Hoffman EP: **Expression profiling in the muscular dystrophies: identification of novel aspects of molecular pathophysiology.** *J Cell Biol* 2000, **151:** 1321-1336.

63. Haslett JN, Sanoudou D, Kho AT, Han M, Bennett RR, Kohane IS *et al*.: **Gene expression profiling of Duchenne muscular dystrophy skeletal muscle.** *Neurogenetics* 2003, **4:** 163-171.

64. Chopard A, Lecunff M, Danger R, Lamirault G, Bihouee A, Teusan R *et al*.: **Large-scale mRNA analysis of female skeletal muscles during 60 days of bed rest with and without exercise or dietary protein supplementation as countermeasures.** *Physiol Genomics* 2009.

65. Gaborit N, Wichter T, Varro A, Szuts V, Lamirault G, Eckardt L *et al*.: **Transcriptional profiling of ion channel genes in Brugada syndrome and other right ventricular arrhythmogenic diseases.** *Eur Heart J* 2009, **30:** 487-496.

66. Lamirault G, Gaborit N, Le MN, Chevalier C, Lande G, Demolombe S *et al*.: **Gene expression profile associated with chronic atrial fibrillation and underlying valvular heart disease in man.** *J Mol Cell Cardiol* 2006, **40:** 173-184.

67. Steenman M, Lamirault G, Le MN, Le CM, Escande D, Leger JJ: **Distinct molecular portraits of human failing hearts identified by dedicated cDNA microarrays.** *Eur J Heart Fail* 2005, **7:** 157-165.

68. Rouger K, Le CM, Steenman M, Potier MC, Gibelin N, Dechesne CA *et al*.: **Global/temporal gene expression in diaphragm and hindlimb muscles of dystrophin-deficient (mdx) mice.** *Am J Physiol Cell Physiol* 2002, **283:** C773-C784.

69. Steenman M, Lamirault G, Le MN, Le CM, Escande D, Leger JJ: **Distinct molecular portraits of human failing hearts identified by dedicated cDNA microarrays.** *Eur J Heart Fail* 2005, **7:** 157-165.

70. Steenman M, Chen YW, Le CM, Lamirault G, Varro A, Hoffman E *et al*.: **Transcriptomal analysis of failing and nonfailing human hearts.** *Physiol Genomics* 2003, **12:** 97-112.

71. Le Meur N, Lamirault G, Bihouee A, Steenman M, Bedrine-Ferran H, Teusan R *et al*.: **A dynamic, web-accessible resource to process raw microarray scan data into consolidated gene expression values: importance of replication.** *Nucleic Acids Res* 2004, **32:** 5349-5358.

72. Tusher VG, Tibshirani R, Chu G: **Significance analysis of microarrays applied to the ionizing radiation response.** *Proc Natl Acad Sci U S A* 2001, **98:** 5116-5121.

73. Smyth GK: **Linear models and empirical bayes methods for assessing differential expression in microarray experiments.** *Stat Appl Genet Mol Biol* 2004, **3:** Article3.

74. Cox TF, Cox MAA: **Multidimensional Scaling - Monographs on Statistics and Applied Probability 88.** Boca Raton (FL, USA): Chapman and Hall/ CRC; 2001.
