# Supplementary material for: Meta-analysis of muscle transcriptome data using the MADMuscle database reveals biologically relevant gene patterns
Source: BMC Genomics. 2011 Feb 16;12:113. doi: 10.1186/1471-2164-12-113 (PMC3049149; doi:10.1186/1471-2164-12-113)
Supplement: Additional File 4 — Re- normalization of MADMuscle data sets. In this supplementary file, we illustrate the re-normalization procedure of each data set to correct remaining bias. [file 1471-2164-12-113-S4.DOC]

**Additional file 4**: **Re- normalization of MADMuscle data sets.**

A LOWESS normalization procedure was applied to correct remaining linear (**A**) and non linear (**B**) bias in all data sets. (**A**) Among the 24 samples of the GEO GSE1004_GPL91 data set, one sample (GSM15832, denoted by an arrow) shows weaker global intensity (raw data in the upper panel) as illustrated by characteristic values of hybridization signals (minimum in red, first quartile in yellow, median in blue, third quartile in pink and maximum in green). This linear bias is corrected by LOWESS normalization (normalized data in the bottom panel). (**B**) Non linear bias can also be detected among samples in high values (often saturation) or in low values (often background effect). The GSM50581 sample from the GEO GSE2629_GPL81 data set shows such a non linear effect in the strongest values (denoted by yellow oval) of hybridization signals (Scatter Plot of the raw sample vs median profile in the upper panel). This local bias was smoothed by LOWESS (Scatter Plot of the normalized sample vs median profile in the middle panel). Notice that non linear effect in low signal values (denoted by an arrow) was also smoothed (Scatter Plot of the raw sample vs normalized sample in the bottom panel) by this procedure.
